# Concurrent CNS tumors and multiple sclerosis: retrospective single-center cohort study and lessons for the clinical management

**DOI:** 10.1007/s10072-022-06142-4

**Published:** 2022-05-19

**Authors:** Yavor Yalachkov, Dilara Dabanli, Katharina Johanna Wenger, Marie-Therese Forster, Joachim P. Steinbach, Martin Voss

**Affiliations:** 1 Department of Neurology, University Hospital Frankfurt, Schleusenweg 2-16, 60528 Frankfurt, Germany; 2 Institute of Neuroradiology, University Hospital Frankfurt, Frankfurt, Germany; 3 Department of Neurosurgery, University Hospital Frankfurt, Frankfurt, Germany; 4 Dr. Senckenberg Institute of Neurooncology, University Hospital Frankfurt, Frankfurt, Germany

**Keywords:** CNS tumor, Multiple sclerosis, Concurrent CNS diseases, Clinical management

## Abstract

**Introduction:**

The concurrent presence of both central nervous system (CNS) tumors and multiple sclerosis (MS) poses various diagnostic and therapeutic pitfalls and makes the clinical management of such patients challenging.

**Methods:**

In this retrospective, single-center cohort study, we searched our clinical databases (2006–2019) for patients with concurrent CNS tumors and MS and described their disease courses. Age at diagnosis of the respective disease and probabilities for MS disease activity events (DAEs) with vs. without prior tumor-specific therapy were tested pairwise using *t*-test for dependent samples and exact binomial test.

**Results:**

*N* = 16 patients with concurrent CNS tumors and MS were identified. MS diagnosis preceded the CNS oncological diagnosis by an average of 9 years (*p* = 0.004). More DAEs occurred in patients without prior chemotherapy (83.3%) than in patients with prior chemotherapy (16.7%; *p* = 0.008). This effect did not reach significance for patients with prior radiation therapy/radiosurgery (66.7% vs. 33.3%, *p* = 0.238). The average interval between DAEs and the last documented lymphopenia was 32.25 weeks.

**Conclusions:**

This study describes the clinical and demographic features of patients with concurrent CNS tumors and MS and suggests several practical approaches to their clinical management. Our findings suggest that adding a disease-modifying MS therapy to the regimen of patients treated with chemotherapy is necessary only if the patient suffers from a highly active, aggressive course of MS. In view of the lack of prospective trials, individual risk assessments should remain the foundation of the decision on MS treatment in concurrent CNS tumor diseases.

## Introduction

Inflammatory autoimmune diseases and tumors of the central nervous system (CNS) can be viewed as two opposite results of neuroimmunological dysfunction: while hyperactivity might lead to primary neuroinflammatory diseases such as multiple sclerosis (MS), immunosuppression promotes the development and progress of brain tumors [1]. Thus, the concurrent presence of both MS and CNS tumors can be rather challenging in clinical practice. Inconclusive diagnostics (e.g., in the case of pseudoprogression) as well as therapeutic pitfalls can severely complicate the management of such patients.

Studies based on general population register data demonstrated a decreased overall cancer risk among patients with MS but an increased risk for brain tumors, which, as the authors suggest, may be due to more frequent neuroimaging in MS patients, i.e., a surveillance bias [2]. Another large study investigated the occurrence of brain tumors in 33 different autoimmune diseases and found that none of them (including MS) influences the risk of glioma development but that autoimmune diseases negatively affect survival in glioma and meningioma patients, probably due to the added physical burden or therapeutic limitations [3]. Accordingly, some of the challenges the clinical management of concurrent CNS tumors and MS pose have been described in the literature but due to the rare coincidence of these two CNS diseases, current evidence is limited to single case reports and, to our best knowledge, no cohort studies have been reported [4–9].

To lay out the main characteristics of the population affected by both diseases, we conducted a retrospective single-center cohort study encompassing patients with concurrent MS and CNS tumors. We focused on characterizing the demographic and clinical variables of this cohort. Furthermore, we evaluated the possible association between tumor therapies and subsequent MS disease activity. This is a particularly relevant question since disease-modifying therapies (DMT) with additional immunosuppressive or immunomodulatory effects can further aggravate the immune system which might have already been compromised by the tumor therapy (e.g., chemotherapy). In this case, adding DMT to an existing chemotherapy might result in an unfavorable risk–benefit ratio, e.g., an increased risk of infection. On the other hand, ongoing MS disease activity without adequate DMT could result in rapidly accumulating neurological deficits that, combined with deficits related to CNS tumors, may severely limit the individual’s quality of life and survival. Finally, we describe three representative cases as examples of the typical challenges in the clinical management of such patients.

## Methods

This single-center retrospective study was approved by the Institutional Review Board (IRB) of the University Hospital Frankfurt (Protocol-Number: SNO-13–2019). It was performed in line with the principles of the Declaration of Helsinki. Consecutive patients who had been registered in the clinical information system of the University Hospital Frankfurt between 2006 and 2019 were identified by filtering for ICD-10 diagnosis codes encompassing MS (G35.1x, G35.2x, G35.3x, G35.9) or unspecified encephalitis, myelitis, or encephalomyelitis (G04.9) and concurrent tumor diseases (CXX.X). Patients had to be seen at least once by a specialist at the Department of Neurology or Dr. Senckenberg Institute of Neurooncology at the University Hospital Frankfurt. Inclusion criteria were (1) diagnosis of CNS tumor (primary CNS tumor or CNS metastasis/meningeal carcinomatosis) and (2) diagnosis of MS (either relapsing–remitting [RRMS], secondary/primary progressive [SPMS/PPMS], or clinically isolated syndrome [CIS]). Patients were excluded if they met only one or none of the two inclusion criteria. The data was carefully reviewed by two different investigators and cases were included only if their evaluation was unequivocal. Inconclusive cases (*n* = 1) were referred to a third senior investigator for a final decision.

For all remaining patients, the following information was extracted: age at MS diagnosis, age at CNS tumor diagnosis, gender, neurooncological diagnosis [10], non-CNS oncological diagnosis (for the patients with CNS metastasis/meningeal carcinomatosis), tumor therapy after neurooncological diagnosis (classified as CNS tumor resection, radiation therapy/radiosurgery, chemotherapy, or molecular/antibody tumor therapy), MS disease phenotype, oligoclonal banding positivity, MS therapy after establishment of neurooncological diagnosis, number and form of MS disease activity events (DAEs, i.e., clinical relapses or radiological signs of MS activity, defined as new/enlarging T2/FLAIR-hyperintense MRI lesions or gadolinium-enhancing T1-weighted MRI lesions), and occurrence of lymphopenia. For consistency purposes, cases with MS diagnosis established using older revisions of the McDonald criteria or where the exact diagnostic criteria had not been referred to, were reviewed by two different investigators and adjusted unequivocally to the latest revision (2017) of the McDonald criteria [11]. Thus, for example, MS cases with recorded relapses but with no history of progression independent of relapse activity were classified as relapsing–remitting MS [11]. For patients with CIS, we ensured that after applying the most recent revision of the McDonald criteria [11], RRMS criteria were still not met.

**Fig. 1:**
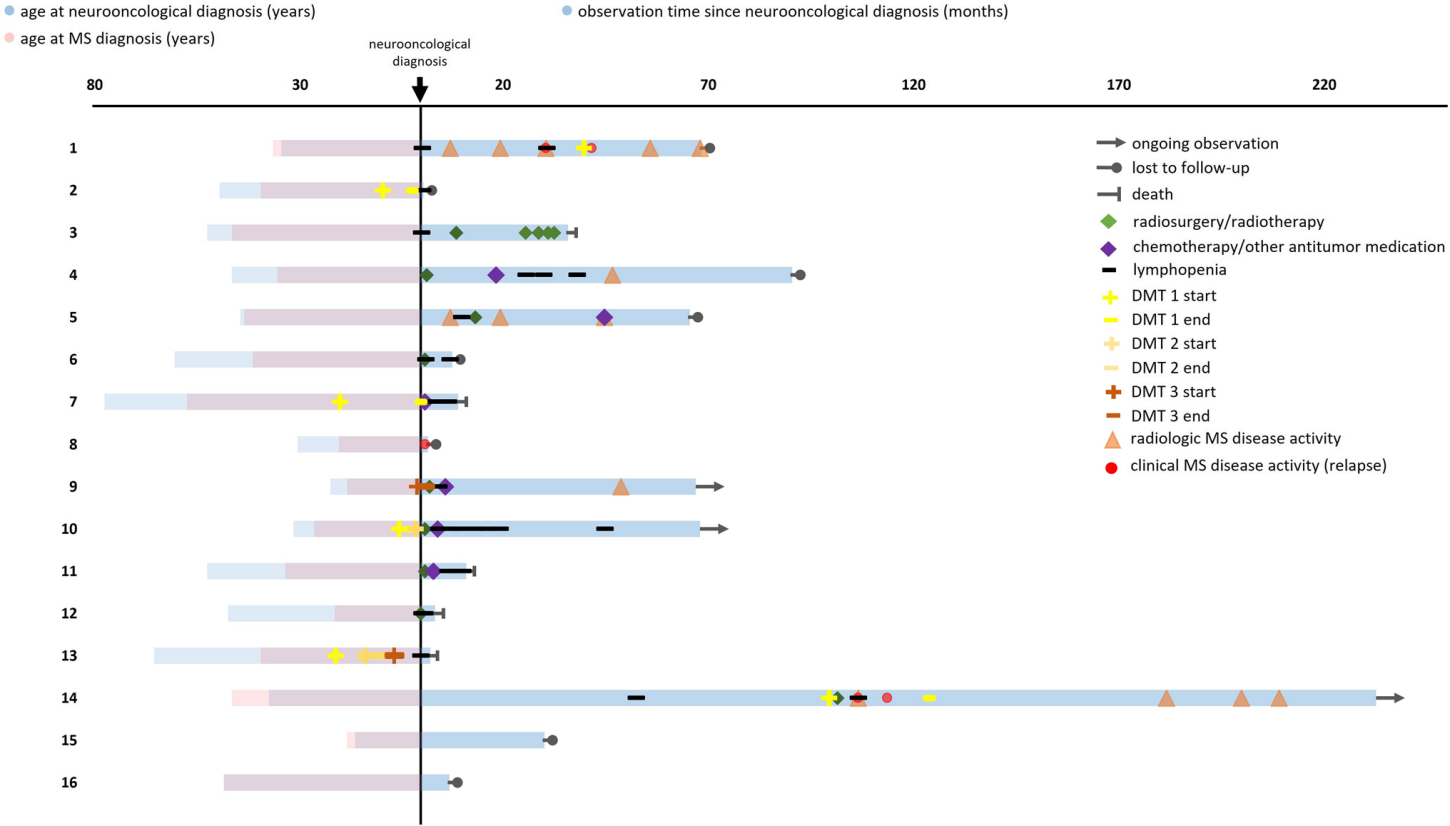
The disease course of concurrent CNS tumor and MS after establishment of the neurooncological diagnosis. To illustrate the course of the two concurrent CNS diseases after the establishment of the neurooncological diagnosis, we employed a swimmer plot. The center of the coordinate system (zero) was defined as the time at which the neurooncological diagnosis was established. On the y-axis, each bar represents a single subject with *n* = 16 patients in total. The x-axis illustrates the time before (left side of the swimmer plot; displays the age [in years] at which the corresponding MS or CNS tumor diagnosis was established) and after (right side of the swimmer plot; displays the common observation time [in months]) the neurooncological diagnosis. On the right side of the swimmer plot, the individual disease course with the corresponding therapies, clinical, imaging, and laboratory findings as well as the end of observation (death/ongoing observation/lost to follow-up) is outlined

To illustrate the course of the two concurrent CNS diseases after the establishment of the neurooncological diagnosis, we employed a swimmer plot (Fig. 1). The origin of the coordinate system (zero) was defined as the point of time when the neurooncological diagnosis had been established. On the y-axis, each bar represents a single subject with *n* = 16 patients in total. The x-axis illustrates the time before (left side of the swimmer plot; displays the age [in years] at which the corresponding MS or neurooncological diagnosis was established) and after (right side of the swimmer plot; displays the common observation time [in months]) the neurooncological diagnosis. On the right side of the swimmer plot, we also outlined the individual disease course with the corresponding therapies, clinical, imaging, and laboratory findings as well as the end of observation (death/ongoing observation/lost to follow-up).

Based on the collected data, we estimated the association between neurooncological therapy and MS disease activity. For this purpose, DAEs were categorized with regard to the presence or absence of (1) prior chemotherapy at any time after the neurooncological diagnosis and (2) prior radiation therapy/radiosurgery at any time after the neurooncological diagnosis. In addition, we determined the interval between each DAE and the last chemotherapy or radiation therapy/radiosurgery as well as the interval between each DAE and the last recorded lymphopenia.

Patients’ age at the time of MS diagnosis and at the time of CNS tumor diagnosis were compared using a *t*-test for dependent samples (two-tailed, significance level set at *p* < 0.05). The proportions of DAEs with vs. without prior chemotherapy and with vs. without prior radiation therapy/radiosurgery were computed and the respective probabilities were estimated and compared pairwise using the exact binomial test (two-tailed, significance level set at *p* < 0.05). Clopper-Pearson 95% confidence intervals were computed correspondingly.

Finally, we describe three representative cases to illustrate some of the diagnostic and treatment pitfalls and offer corresponding strategies which may be employed in the clinical management of these patients.

## Results

The search for concurrent MS/encephalitis/myelitis/encephalomyelitis and tumor ICD-10 diagnosis codes retrieved *n* = 83 patients. After a careful examination of their clinical data, diagnostic work-up, and final diagnoses, *n* = 67 patients did not meet the study inclusion criteria. *N* = 16 patients with concurrent CNS tumor and MS were included. The demographic and clinical features of the sample as well as the results from the statistical testing are presented in Table 1.

**Table 1:** Demographic and clinical information

	***n***	**%**				
Gender						
Female	9	56.3				
Male	7	43.7				
Neurooncological diagnosis						
CNS lymphoma	1	6.25				
CNS metastases/meningeal carcinomatosis	5	31.25				
Glioblastoma	2	12.5				
Astrocytoma/anaplastic astrocytoma	2	12.5				
Pilocytic astrocytoma	1	6.25				
Ependymoma/subependymoma, *n* (%)	2	12.5				
Glioma (unspecified)	1	6.25				
Meningioma	2	12.5				
Non-CNS oncological diagnosis						
Mammary carcinoma	1	6.25				
Lung carcinoma	3	18.75				
Melanoma	1	6.25				
Other oncological diagnosis, not CNS relevant						
Mammary carcinoma	1	6.25				
Mantle cell lymphoma	1	6.25				
CNS tumor resection						
No	7	43.75				
Yes	8	50.0				
Information not available	1	6.25				
Radiation therapy/radiosurgery after neurooncological diagnosis						
No	6	37.5				
Yes	10	62.5				
Chemotherapy after neurooncological diagnosis						
No	10	62.5				
Yes	6	37.5				
Molecular/antibody tumor therapy after neurooncological diagnosis						
No	14	87.5				
Yes	2	12.5				
MS disease phenotype						
RRMS	13	81.25				
SPMS	1	6.25				
CIS	2	12.5				
Oligoclonal banding positivity						
No	1	6.25				
Yes	9	56.25				
Information not available	6	37.5				
MS therapy after neurooncological diagnosis						
None	9	56.25				
Continued/started	7	43.75				
Interferon beta-1a/b	2	12.5				
Glatiramer acetate	1	6.25				
Dimethyl fumarate	2	12.5				
Repeated intervals of high-dose corticosteroid	1	6.25				
No information available	1	6.25				
MS therapy stopped after neurooncological diagnosis						
Interferon beta-1a/b	1	6.25				
Azathioprine	1	6.25				
Teriflunomide	1	6.25				
Natalizumab	1	6.25				
			*p* value	Estimate	95% CI	
					Lower	Upper
MS disease activity event (DAE)						
Clinical relapse events (number of patients)	5 (3)	27.8 (18.8)	0.096^d^	0.722^a^	0.465	0.903
Radiologic activity events (number of patients)	13 (5)	72.2 (31.3)				
Number of patients with any DAE	6	37.5				
Chemotherapy preceding DAE						
No	15	83.3	0.008^d^	0.167^b^	0.036	0.414
Yes	3	16.7				
Radiation therapy/radiosurgery preceding DAE						
No	12	66.7	0.238^d^	0.333^c^	0.133	0.590
Yes	6	33.3				
	Mean	SD				
Age (years) at						
Neurooncological diagnosis	44.38	15.81	0.004^e^			
MS diagnosis	35.38	11.86				
Interval between DAE and chemotherapy (weeks)	23.67	21.83				
Interval between DAE and radiation therapy/radiosurgery (weeks)	24.33	19.22				
Interval between DAE and last lymphopenia (weeks)	32.25	35.70				
Total observation time (months)						
All participants (median, IQR)	21 (61)					
Chemotherapy preceding DAE	67 (12)					
No chemotherapy preceding DAE	68 (115)					

Number of patients and proportion (in % of the whole sample) are presented for the most relevant demographic and clinical categories. *CNS*, central nervous system; *MS*, multiple sclerosis; *RRMS*, relapsing–remitting MS; *SPMS*, secondary progressive MS; *CIS*, clinically isolated syndrome; *DAE*, disease activity event (i.e., either a clinical relapse or radiologic activity). ^a^Probability for DAE; ^b^estimated probability for chemotherapy/antibody/molecular therapy preceding DAE; ^c^estimated probability for radiotherapy/radiosurgery preceding DAE; ^a, b, c^ were computed using the exact binomial test and the corresponding exact Clopper-Pearson 95% confidence interval; ^d^exact binomial test was used to test for a significant deviation of the observed from the expected probability of 0.5 for the respective category; ^e^*t*-test for dependent samples

The majority of the patients (56.25%) were female. MS diagnosis preceded the first CNS oncological diagnosis by an average of 9 years (mean age 35.38 vs. 44.38, *t*-test for dependent samples, *p* = 0.004). The majority of MS phenotypes were RRMS or CIS (93.75%) and the majority of the CNS tumors were metastases/meningeal carcinomatosis (31.25%). Other CNS tumors were distributed more evenly (glioblastoma, astrocytoma/anaplastic astrocytoma, ependymoma/subependymoma, and meningioma each 12.5%, while pilocytic astrocytoma and unspecified glioma were represented with 6.25% each). Most of the metastases originated from lung carcinoma (18.75% of all patients). Other primary cancers were mammary carcinoma or melanoma (each 6.3% of all patients). Approximately half of the patients (50%) underwent CNS tumor resection. The majority of the patients (62.5%) were treated with radiation therapy/radiosurgery. Fifty percent received chemotherapy (temozolomide; R-CHOP (rituximab, cyclophosphamide, doxorubicin, vincristine, prednisolone), R-MTX (rituximab, methotrexate)) or molecular/antibody therapy following the neurooncological diagnosis. 56.25% were not treated with a disease-modifying MS therapy after the establishment of the neurooncological diagnosis, while 43.75% started or continued their MS therapy after CNS tumor diagnosis. Twenty-five percent of the patients discontinued MS therapy after the neurooncological diagnosis had been established.

Most of the DAEs (72.2%) were signs of radiological activity in brain/spinal cord imaging. Only 27.8% of the events were clinically apparent relapses. DAEs affected in total 37.5% of the patients. More DAEs occurred in patients without than in patients with prior chemotherapy (83.3% vs. 16.7%; exact binomial test, *p* = 0.008). This effect did not hold true for patients with prior radiation therapy/radiosurgery treatment (66.7% vs. 33.3%, exact binomial test, *p* = 0.238). Average intervals between each type of tumor therapy (chemotherapy and radiation therapy/radiosurgery) and the next DAE were almost identical (23.67 vs. 24.33 weeks). The average interval between DAEs and the last documented lymphopenia was 32.25 weeks.

## Discussion

In this retrospective, single-center cohort study, we describe a series of patients with concurrent CNS tumor and MS diagnoses.

Not surprisingly, some of the patients discontinued or never started their MS therapy (56.25%) after establishment of the neurooncological diagnosis. A possible explanation could be the low to moderate average level of MS disease activity after CNS tumor diagnosis (only 37.5% of our patients were affected by DAEs) with a risk–benefit ratio that did not favor DMT. Furthermore, discontinuing MS therapy which had been initiated before the neurooncological diagnosis might be related to concerns about DMT-associated immunosuppression and increased risk of infection. Further concerns could be related to a possible risk of neoplasm, which has been reported for some DMTs among MS patients. Although Grytten et al. reported an increased incidence of cancer among MS patients compared to controls for the time period from 1996 to 2017, corresponding in time to the introduction of DMT for MS, this effect has been observed predominantly among MS patients older than 60 years of age [12] and other studies analyzing similar time periods could not confirm these findings but reported instead no increased incidence of malignancy, neither in comparison with the general population nor in relation to DMT [13, 14]. A recent meta-analysis with meta-regression by Papadopoulos et al. found that treatment with DMT did not modify the risk of neoplasms in MS clinical trials from 1991 to 2020, which may reflect a low carcinogenic potential of DMTs and/or that the neoplasia latencies far exceed the typical MS trial observation periods [15]. 43.75% of our patients started or kept their MS therapy after tumor diagnosis, which might have been motivated by the concern of ongoing MS disease activity.

Interestingly, DAEs defined as clinically apparent relapses or radiological signs of MS disease activity occurred mainly in patients without prior chemotherapy for CNS tumors (83.3% of all DAEs). On the contrary, only a very small proportion of DEAs (16.7%) occurred at any time after chemotherapy for CNS tumor diagnosis, which suggests that chemotherapy reduces MS disease activity via its immunosuppressive mechanisms of action. This is consistent with the fact that some DMTs have similar mechanisms of action or even have been derived from therapeutics used in cancer treatment [16]. Moreover, these findings imply that chemotherapy employed for the treatment of CNS tumors reduces by far the need for additional DMT in concurrent MS and that there might not be a significant level of MS disease activity after chemotherapy. DAEs in patients with prior chemotherapy were seen on average 23.67 weeks (i.e., approximately 6 months) after chemotherapy, which corresponds to the time interval of treatment in, e.g., some B-cell depleting therapies. Interestingly, the average interval between DAEs and the last reported lymphopenia was on average 8 months, which also roughly corresponds to this interval. Therefore, recurring DAEs in patients with preceding chemotherapy might be related to the repopulation of immune cells and the resulting reconstitution of the immune system. Finally, patients with preceding chemotherapy and absent DAEs were observed for more than 6 years on average (median 68 months, Table 1), which is a reasonable time for a follow-up to assess the effect of a particular treatment on MS disease activity. Our findings suggest long-lasting effects of the chemotherapy on the immune system and imply that patients with concurrent MS and CNS tumors benefit from DMT only if they do not receive additional chemotherapy. Vice versa, chemotherapy treatment might be associated with an unfavorable risk–benefit ratio of an additional DMT unless the patient has a highly active, very aggressive course of MS. Thus, individual risk assessments should be the foundation of the decision on the MS treatment approach in concurrent CNS tumor diseases.

Interestingly, we did not find evidence for an altered ratio of DAEs after radiation therapy or radiosurgery treatment. There have been reports on increased radiosurgery toxicity in MS [17] and radiotherapy might stimulate the immune response [18]. Most interestingly, a case report described an MS-like disease confined to the irradiated part of the cervico-thoracic spinal cord of a patient treated before with radiation therapy for Hodgkin’s lymphoma, suggesting that alterations of antigenic make-up may enable MS-specific autoimmune attacks by a pre-existent immunological mechanism [19]. However, at least in this small sample, no deterioration of MS followed radiotherapy. Thus, radiation therapy or radiosurgery treatment did not seem to affect MS disease activity.

There are several limitations to our study. Due to the rare prevalence of the concurrent presence of the two diseases, the sample size was limited. However, most of the baseline demographical characteristics of our sample were consistent with the characteristics of the respective disease population (CNS tumor vs. MS). While the mean age at MS diagnosis worldwide is 32 years [20], most of adult gliomas occur in patients aged 45 years or older, [21] and the incidence of brain metastases is typically higher in older patients [22]. Consistently, our results showed a similar difference between the age at which the MS (35.38 years) and the CNS tumor (44.38 years) diagnoses were established. Most of the reported patients had a RRMS or CIS diagnosis, which corresponds to previously reported distributions of MS disease phenotypes [23]. However, we cannot rule out a bias related to the more frequent MRI follow-ups in relapsing MS phenotypes, especially if patients switch treatments and need a new baseline MRI or are at risk of serious treatment-related adverse events and need to be monitored more frequently [24]. Frequent MRI follow-ups increase the probability of detecting CNS neoplasia. On the other hand, symptoms in progressive MS are typically advancing more slowly than relapse-associated worsening in active MS and thus it is at least theoretically possible that CNS tumor occurrence might be mistakenly considered as a MS-related progress in SPMS/PPMS patients, which might delay the initiation of new brain/spinal imaging and corresponding diagnostics. Last but not least, due to the retrospective nature of this study, not all of the relevant variables were available—for example, EDSS scores were missing for many of the subjects and were therefore not included in the analysis. Visitations had been scheduled purely out of clinical indication and consequently the intervals between the single observations were not standardized. Exact time points of antiedematous treatment with dexamethasone were not documented in the medical records. In some cases, corticosteroids treatment might have reduced or even suppressed ongoing MS disease activity. Future prospective cohort studies should include these aspects. Similarly, the inclusion of patients treated with checkpoint-inhibitors in prospective studies would be highly interesting from both clinical and pathophysiological perspectives. Another limitation was that due to the small sample size we were not able to differentiate between the effectivity of the different chemotherapies in suppressing MS disease activity. This would be an intriguing question since some therapies may affect autoimmunity to a different extent and its answer could guide a more precise MS immunotherapy in patients treated with chemotherapy.

**Fig. 2:**
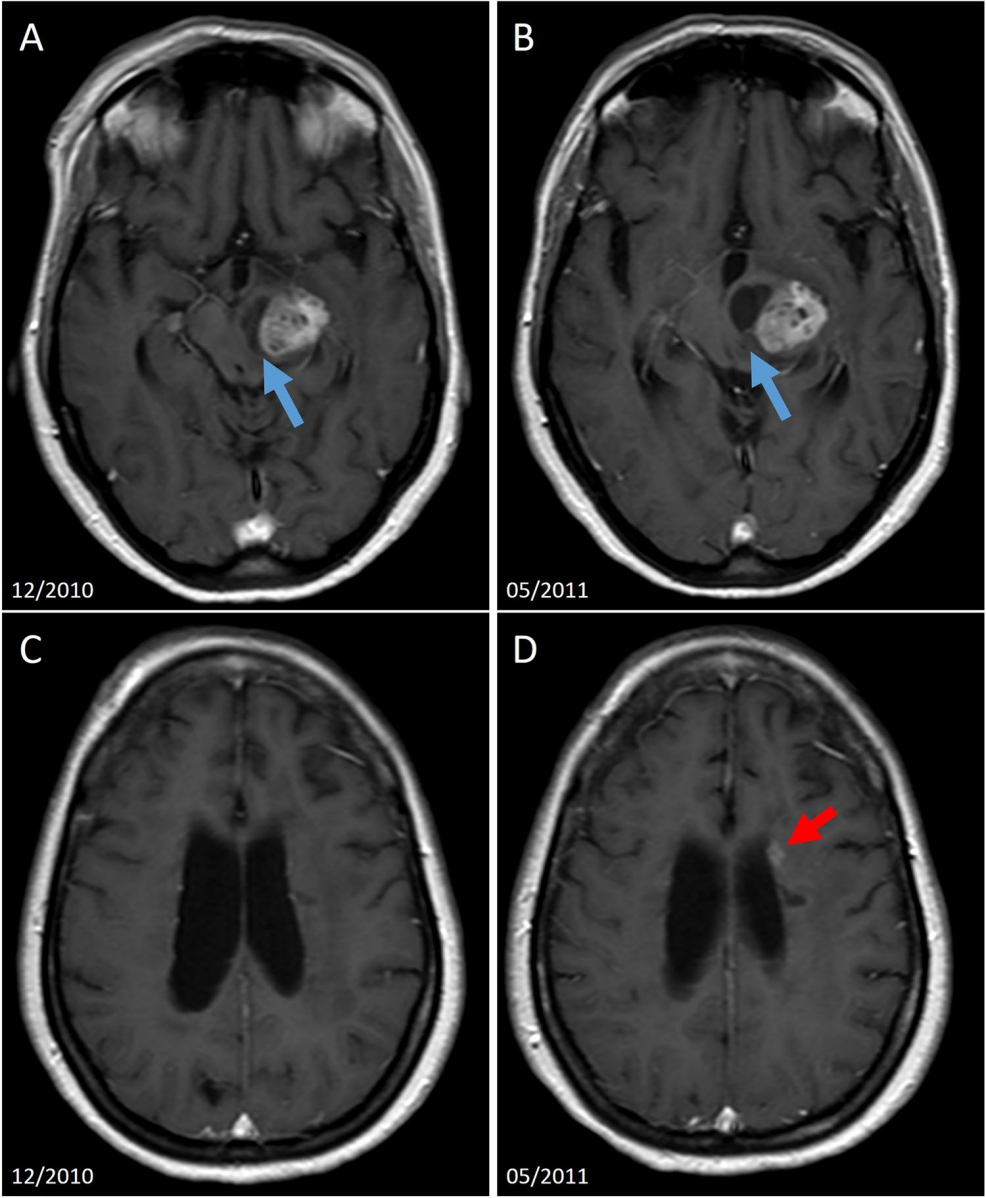
Pseudoprogression in concomitant MS and CNS tumor. **A** This 46-year-old female patient was diagnosed with a pilocytic astrocytoma affecting the left thalamus and the left cerebral peduncle. Relapsing–remitting MS had been diagnosed 11 years before that. She was treated with radio- and chemotherapy for the astrocytoma. During one of the 6-month follow-ups, she reported new dysarthria and increasingly impaired ambulation. Brain MRI revealed that the cystic portion of the tumor has grown (**A**–**B**, blue arrows) affecting more strongly the left cerebral peduncle. Additionally, a new periventricular gadolinium-enhancing MS lesion has appeared, which was not seen on the previous brain MRI (**C**–**D**, red arrow). Because of increasingly pronounced symptoms and further growth of the cystic portion, the cystic portion was punctured. No corticosteroid therapy was initiated for the new MS lesion. The lesion was not present on the further follow-ups anymore (not shown here)

**Fig. 3:**
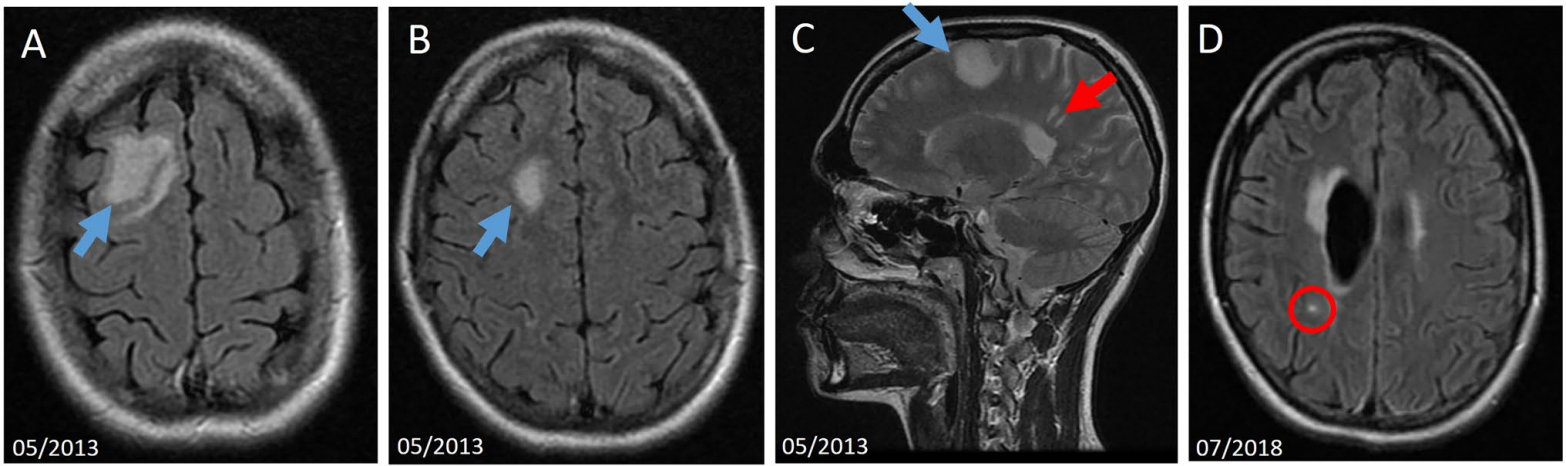
Resuming DMT in concomitant MS and CNS tumor. **A**–**C** This 21-year-old female patient presented for a second opinion at our hospital. She had been diagnosed 4 years before that with a relapsing–remitting multiple sclerosis (MS). During the diagnostic process, a right frontal lesion not typical for MS was detected (indicated by blue arrows; the red arrow shows two exemplary MS lesions). During the last follow-ups, the right frontal lesion has been expanding slowly. Therefore, primary resection was recommended and done 14 months after the first presentation in our clinic, leading to the diagnosis of anaplastic astrocytoma and followed by radio- and chemotherapy. **D** Four years later, the patient presented with a new numbness of the right arm. There were no signs of a tumor recurrence. A new right hemispheric T2/FLAIR-hyperintense lesion was detected on the brain MRI (red circle). No spinal MRI was done. Based on the new clinical relapse and radiologic signs of activity, the resumption of a disease-modifying therapy (DMT) for the MS, which had been stopped before the chemotherapy, was recommended

**Fig. 4:**
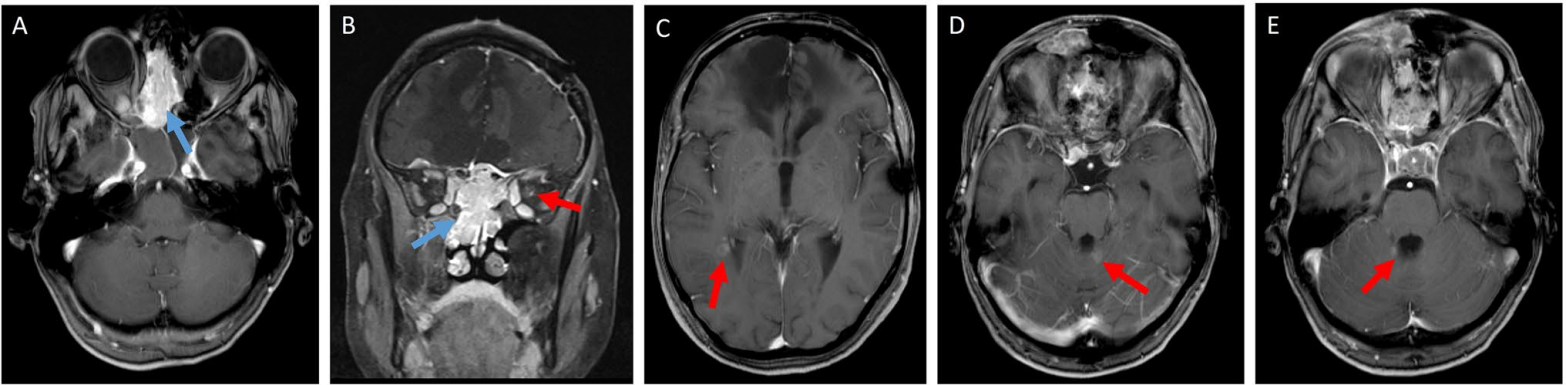
Equivocal clinical symptoms in concomitant MS and CNS tumor. This 46-year-old female patient has been diagnosed with multiple sclerosis and recurrent multifocal meningioma. The latter has been resected surgically twice and treated with a radiation therapy after the last detection of tumor growth. Three months after the radiation therapy the patient presented again with a vision loss in the left eye. Optic nerve compression by a new tumor growth was suspected. However, the patient reported also new general weakness, numbness in all four extremities, unsteady gait and clinically a sixth nerve palsy on the right side was detected. Brain MRI did not show any new tumor growth (**A**–**B**, blue arrows) but revealed several new gadolinium-enhancing MS lesions including one in the optic nerve (**C**–**E**, red arrows). Corticosteroids resulted in improvement of all symptoms, but the vision loss progressed further. Plasmapheresis was initiated and resulted in a partial improvement of vision after 9 cycles. Radiogenic damage to the optic nerve was also considered as an alternative diagnosis but the effect of plasmapheresis suggested inflammatory MS activity

Based on our observations of the clinical courses of this exclusive cohort, we would like to outline the following lessons for the clinical management of concurrent CNS tumor and MS diseases: First, in patients treated with cytotoxic chemotherapy, there is little to no evidence for an “add-on” DMT for MS, since the tumor-specific therapy results in a sufficient suppression of the immune system. DMT could be considered, however, as our data suggests, 6–8 months after the last tumor-specific treatment and/or lymphopenia, especially in the case of clinical and/or imaging findings indicative of ongoing MS disease activity. Particularly in patients who exhibit a highly active MS disease course and are treated for their CNS tumor disease solely with radiotherapy, maintaining or starting DMT seems reasonable. Second, in some cases methylprednisolone treatment of relapses might not be necessary and instead watchful waiting might suffice, especially if the main reason for the clinical worsening can be clearly identified as related to the CNS tumor activity (Fig. 2). On the other hand, if clinical and radiological signs of disease activity persist, DMT initiation/resumption can be recommended to reduce the risk for accumulating disability (Fig. 3). Cases with one of the diseases mimicking the other are particularly challenging. In such cases, the clinician might be tempted to explain the appearance of new or worsening of already present symptoms by the disease, which is currently given priority in treatment. However, symptoms which are not fully and reliably explained by the respective disease should always prompt diagnostics and further evaluation since this might have important therapeutic consequences, as our experience suggests (Fig. 4).

Our study describes the clinical and demographic features of patients with concurrent CNS tumors and MS and introduces several practical approaches to their clinical management. Our findings suggest that adding a disease-modifying MS therapy to the regimen of patients treated with cytotoxic chemotherapy is associated with an unfavorable risk–benefit ratio, unless the patient suffers from a highly active, aggressive course of MS. Decisions on clinical management of concurrent CNS tumor and MS should therefore always be based on an individual risk assessment.

## Data Availability

The data that support the findings of this study are available in anonymized form from the corresponding author upon reasonable request. The data are not publicly available due to privacy and ethical restrictions.
